# Thermoelectric properties of ballistic Normal–Weyl semimetal-Normal junction

**DOI:** 10.1038/s41598-023-41355-3

**Published:** 2023-08-31

**Authors:** Jafar Lotfi, Babak Abdollahipour

**Affiliations:** Faculty of physics, University of Tabriz, 51666-16471 Tabriz, Iran

**Keywords:** Electronic properties and materials, Thermoelectric devices and materials, Topological insulators

## Abstract

Weyl semimetals are a new class of topological materials possessing outstanding physical properties. We investigate the thermoelectric properties of a ballistic Weyl semimetal specimen connected to two normal contacts. We introduce a model to evaluate the thermoelectric coefficients of the junction and analyze its features along two distinct directions, one along the chiral axis of the Weyl semimetal and the other perpendicular to it. We demonstrate that the thermoelectric response of this junction depends on whether it is along the chiral axis of the Weyl semimetal or not. Electrical and thermal conductances of this junction reveal considerable dependence on the length and chemical potential of the Weyl semimetal layer. In particular, we observe that, decreasing the chemical potential in the normal contacts enhances the Seebeck coefficient and thermoelectric figure of merit of the junction to substantial values. Hence, we unveil that a ballistic junction of Weyl semimetal can serve as a fundamental segment for application in future thermoelectric devices for thermal energy harvesting.

## Introduction

Weyl semimetals (WSMs) are a new class of topological matter that have recently attracted an immense interest^1^. The conduction and valance bands in the energy dispersion of WSMs touch each other at even number of Weyl nodes and have linear dispersions around them^2,3^. The number and chirality of Weyl nodes are specified by symmetry class of the material^4^. WSMs are categorized into type-I^5^ and type-II^6^ depending on whether they have a point like or open Fermi surfaces around the Weyl nodes. Some novel and exotic phenomena such as chiral anomaly^7^, anomalous Hall effect^8,9^, negative magnetoresistance^10^, and anomalous Nernst effect^11^ has been observed in WSMs.

Heat is dissipated in most of the devices and is mainly wasted or caused to overheating the device leading to interference in its functionality. Thermoelectric effects (TEs) are promising for renewable energy harvesting and sorting out energy waste in devices via the heat-voltage conversion, as well for other applications such as thermometry, refrigeration^12,13^. Thermoelectric materials with high thermoelectric efficiency can convert waste heat into useful electricity^14,15^. The efficiency of a system to generate electrical power from a temperature gradient is determined by thermoelectric coefficients^16^. The Seebeck coefficient specifies a current (closed boundary condition) or a bias (open boundary condition) which is induced due to the temperature difference maintained between two reservoirs connected to the system^17,18^. The Nernst coefficient, or transverse Seebeck coefficient, determines the thermally induced current (bias) generated in the direction transverse to both the temperature gradient and the applied magnetic field^19^. Identifying materials with high thermoelectric responses is crucial for developing novel electric generators and coolers. In addition, thermoelectric coefficients provide information about the flow of energy and charge due to the high impact of the density of states on thermodynamic coefficients than the electrical conductance^20–22^. Therefore, investigating TEs can pave as a robust implement for exploration of the system dynamics.

Electronic contribution to the thermal conductivity and the thermopower of WSMs and Dirac semimetals (DSMs) has been studied using a semiclassical Boltzmann approach^23^. It was found that the thermal conductivity and thermopower have an exciting dependence on the chemical potential which is characteristic of the linear electronic dispersion of these materials^24^. It has been shown that these materials have very singular behavior at zero doping and zero temperature due to a quantum anomaly. The thermopower and the thermoelectric figure of merit of DSMs and WSMs subjected to a quantizing magnetic field grows linearly with the field without saturation and can reach extremely high values^25,26^. The impact of the Berry curvature and orbital magnetization on the thermopower in tilted WSMs has been investigated^27^. It was found that the tilt of Weyl nodes induces linear magnetic field terms in the conductivity and thermopower matrices. The linear-B term appears in the Seebeck coefficients when the B-field is applied along the tilt axis. Nernst effect in DSMs and inversion asymmetric WSMs has been calculated within the semiclassical Boltzmann approach^28^. It was found that at the Dirac points, the low temperature and low magnetic field Nernst response is dominated by anomalous Nernst effect, arising from a non-trivial profile of Berry curvature on the Fermi surface. Moreover, the anomalous Nernst and thermal Hall effects in a linearized low-energy model of tilted WSMs have been studied^29–32^.

To the best of our knowledge, there is no investigation about the thermoelectric properties of ballistic junctions composed of WSMs. Here we propose studying the thermoelectric characteristics of a ballistic junction consisting of a WSM layer connected to two normal contacts. We introduce a model to derive the thermoelectric properties of this junction along two perpendicular directions characterizing the band structure of WSM. We find highly direction-dependent electrical and thermal conductances for this junction. However, the Seebeck coefficient of this junction displays slight direction dependence only at low chemical potentials of the leads. Moreover, we demonstrate that this junction acquires high values of the Seebeck coefficient and thermoelectric figure of merit at vanishingly small chemical potentials of the normal leads.

The remnant of the paper is organized as follows. In “Theoretical model and equations”, we present a theoretical model and equations for calculating TEs for the considered structure. “Results and discussions ” is devoted to representing and discussing the main results of this study involving the investigation of the electrical and thermal conductances and Seebeck coefficient in terms of the junction parameters. Eventually, a conclusion is given in “Conclusion”.

## Theoretical model and equations

**Figure 1:**
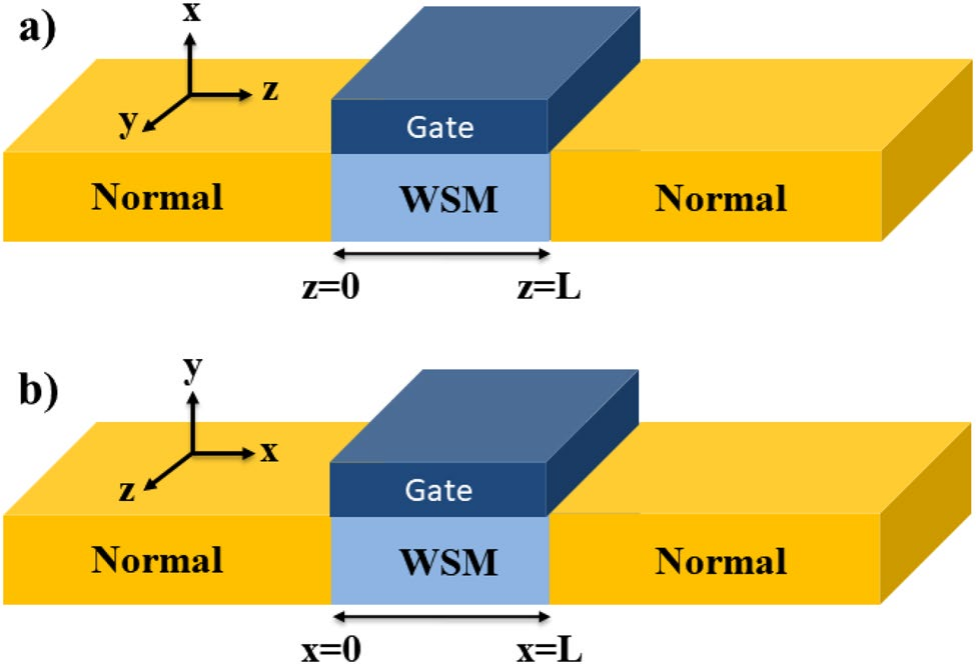
Schematic representation of the considered junctions. (**a**) Junction is along the *z* axis and parallel to the line connecting two Weyl nodes (the chiral axis) of WSM in the momentum space. (**b**) It is along the *x* axis and perpendicular to the chiral axis of WSM.

We consider a ballistic junction composed of a WSM layer with length *L* sandwiched between two semi-infinite normal contacts as shown in Fig. 1. We suppose that the chemical potential in WSM layer can be adjusted via doping or gate voltage. Our aim is investigation of the electronic contribution to the thermoelectric properties of this junction such as electrical conductance (*G*), electronic contribution to thermal conductance ($\kappa _{el}$) , and Seebeck coefficient (*S*). To take into account the asymmetric properties of this junction we study two distinct cases, one when junction is along the *z* axis (the chiral axis) and the second case is when junction is along the *x* axis (perpendicular to the chiral axis) as depicted in Fig. 1a,b, respectively. We consider a minimal model Hamiltonian to describe inversion symmetric WSMs in the full range of energy^33,34^,

1 $$\begin{aligned} H_{WSM}=-\mathcal {M}(k_x^2+k_y^2+k_z^2-k_0^2)\sigma _z+\gamma (k_x\sigma _x+k_y\sigma _y)+\mu _W \sigma _0, \end{aligned}$$

where $k_{x,y,z}$ represent the components of the wave vector, $\sigma _{0}$ is the $2*2$ unit matrix, $\sigma _{x,y,z}$ are the Pauli’s matrices and $\mu _W$ indicates the electrochemical potential. In this model Hamiltonian $\mathcal {M}, \gamma , k_{0}>0$ are parameters that are determined through the experimental or ab-initio calculation results. In this model $k_z=\pm$ $k_{0}$ denote the location of the two Weyl nodes in the momentum space. This minimal model gives a generic description of a pair of Weyl nodes with opposite chirality and, hence, all the topological properties of the inversion symmetric WSMs. In contrast, in the case of the time-reversal symmetric WSMs, a minimal model should support at least four Weyl nodes as two time-reversed pairs of nodes. In a ballistic sample with no scattering between the nodes, two pairs of time-reversed nodes treat independently. The only difference between these pairs is the energy shift relative to each other. Hence, the present model describes the contribution of each of these pairs very well, and by some reflection, it is possible to find the total result. The normal contacts can be described by a simple parabolic energy dispersion. Therefore, the following Hamiltonian is assumed to describe the normal contacts,

2 $$\begin{aligned} H_{N}=-\mathcal {M}(k_x^2+k_y^2+k_z^2)\sigma _0+\mu _N \sigma _0, \end{aligned}$$

where $\mu _N$ represents the electrochemical potential in the normal contacts. We use scattering approach to calculate the thermoelectric coefficients of the considered junctions. Solving the Hamiltonian given by Eq. (1), gives eigenvalues and eigenvectors corresponding to WSM as follows,

3 $$\begin{aligned}{} & {} E_{W}=\pm \sqrt{\varepsilon _k^2+\gamma ^2(k_x^2+k_y^2)}+\mu _W, \end{aligned}$$

4 $$\begin{aligned}{} & {} \psi _{W,1}=\left( \begin{array}{c} 1 \\ u \\ \end{array}\right) e^{i\mathbf {k_{W}}.\textbf{r}}, \psi _{W,2}=\left( \begin{array}{c} v \\ 1 \\ \end{array}\right) e^{i\mathbf {k_{W}}.\textbf{r}}, \end{aligned}$$

where we have defined $\varepsilon _k=\mathcal {M}(k_x^2+k_y^2+k_z^2-k_0^2)$, $k_W$ is the wave vector obtained from the eigenvalue equation Eq. (3), *u* and *v* are given by the following relations,

5 $$\begin{aligned} u=\gamma (k_x-ik_y)/(E_W-\varepsilon _k), \nonumber \\ v=\gamma (k_x+ik_y)/(E_W+\varepsilon _k). \end{aligned}$$

The eigenvalue and corresponding eigenvectors in the normal region are given by,

6 $$\begin{aligned}{} & {} E_{N}=\mathcal {M}(k_x^2+k_y^2+k_z^2)+ \mu _N, \end{aligned}$$

7 $$\begin{aligned}{} & {} \psi _{N,1}=\left( \begin{array}{c} 1 \\ 0 \\ \end{array}\right) e^{i\textbf{k}.\textbf{r}}, \psi _{N,2}=\left( \begin{array}{c} 0 \\ 1 \\ \end{array}\right) e^{i\textbf{k}.\textbf{r}}. \end{aligned}$$

where $\textbf{k}=(k_x, k_y, k_z)$ is the wave vector in the normal region obtained from Eq. (6). Now, we can set up the scattering problem for an electron incident from the left side of the junction. We aim to calculate the properties of the junction in two perpendicular directions. First, we assume that the junction direction be along the *z* axis. We can express the wave function in the left normal ($z<0$) for an electron incident in the first or second states respectively as follows,

8 $$\begin{aligned} \Psi _{L,1}=\left\{ \left( \begin{array}{c}1 \\ 0\end{array} \right) e^{ik_{L,z}z}+r_{1,1}\left( \begin{array}{c}1 \\ 0\end{array} \right) e^{-ik_{L,z}z}+ r_{2,1}\left( \begin{array}{c}0 \\ 1\end{array} \right) e^{-ik_{L,z}z}\right\} e^{i(k_{x}x+k_{y}y)},\nonumber \\ \Psi _{L,2}=\left\{ \left( \begin{array}{c}0 \\ 1\end{array} \right) e^{ik_{L,z}z}+r_{1,2}\left( \begin{array}{c}1 \\ 0\end{array} \right) e^{-ik_{L,z}z}+ r_{2,2}\left( \begin{array}{c}0 \\ 1\end{array} \right) e^{-ik_{L,z}z}\right\} e^{i(k_{x}x+k_{y}y)}, \end{aligned}$$

where $k_{L,z}$ is the *z* component of the wave vector in the left normal, $r_{1,1}$, $r_{2,1}$, $r_{1,2}$ and $r_{2,2}$ describe reflection amplitudes into the first and second states when incident electron is in the first or second states, respectively. In the WSM region ($0\le z \le L$) the wave function reads,

9 $$\begin{aligned} \begin{array}{c} \Psi _{W}=\left\{ g\left( \begin{array}{c}1 \\ v_{+}\end{array} \right) e^{ik_{+,z}z}+f\left( \begin{array}{c}1 \\ v_{-}\end{array} \right) e^{ik_{-,z}z} +p\left( \begin{array}{c}u_{+} \\ 1\end{array} \right) e^{ik_{+,z}z}+q\left( \begin{array}{c}u_{-} \\ 1\end{array} \right) e^{ik_{-,z}z}\right\} e^{i(k_{x}x+k_{y}y)}, \end{array} \end{aligned}$$

where *g*, *f*, *p*, *q* are unknown coefficients, and solutions of *z* component of the wave vector are derived from the eigenvalue relation of WSM region given by Eq. (3) as follows,

10 $$\begin{aligned} k_{\pm ,z}=\sqrt{k_0^2-k_x^2-k_y^2\pm \sqrt{\left( \frac{E-\mu _W}{\mathcal {M}}\right) ^2 -(k_x^2+k_y^2)\left( \frac{\gamma }{\mathcal {M}}\right) ^2}}. \end{aligned}$$

Finally, the wave function in the right normal ($z>L$) for incident electron in the first and second states respectively are given by,

11 $$\begin{aligned} \Psi _{R,1}=\left\{ t_{1,1}\left( \begin{array}{c}1 \\ 0\end{array} \right) e^{ik_{R,z}z}+t_{2,1}\left( \begin{array}{c}0 \\ 1\end{array} \right) e^{ik_{R,z}z}\right\} e^{i(k_{x}x+k_{y}y)},\nonumber \\ \Psi _{R,2}=\left\{ t_{1,2}\left( \begin{array}{c}1 \\ 0\end{array} \right) e^{ik_{R,z}z}+t_{2,2}\left( \begin{array}{c}0 \\ 1\end{array} \right) e^{ik_{R,z}z}\right\} e^{i(k_{x}x+k_{y}y)}, \end{aligned}$$

where $k_{R,z}$ is the *z* component of the wave vector in the right normal. $t_{1,1}$, $t_{2,1}$, $t_{1,2}$ and $t_{2,2}$ express the transmission amplitudes to the first and second states in the right normal when the incident electron is in the first and second states, respectively. To calculate the transmission coefficients, we apply the following boundary conditions which guaranties the particle current conservation,

12 $$\begin{aligned} \begin{array}{c} {\Psi _{W}}\mid _{z=0}={\Psi _{L}}\mid _{ z=0},~~~\hat{v}_{W,z}{\Psi _{W}}\mid _{z=0}=\hat{v}_{N,z}{\Psi _{L}}\mid _{z=0},\\ \\ {\Psi _{W}}\mid _{z=L}={\Psi _{R}}\mid _{z=L},~~~\hat{v}_{W,z}{\Psi _{W}}\mid _{z=L}=\hat{v}_{N,z}{\Psi _{R}}\mid _{z=L}.\\ \end{array} \end{aligned}$$

where $\hat{v}_z=\partial H/\partial k_z$ is the velocity operator along the *z* direction. Eventually, the transmission probabilities are defined according to the following relations,

13 $$\begin{aligned} \begin{array}{c} T_{1,1}=\frac{k_{R,z}}{k_{L,z}}t_{1,1} t_{1,1}^{*},~~~T_{2,1}=\frac{k_{R,z}}{k_{L,z}}t_{2,1} t_{2,1}^{*},\\ \\ T_{1,2}=\frac{k_{R,z}}{k_{L,z}}t_{1,2} t_{1,2}^{*},~~~T_{2,2}=\frac{k_{R,z}}{k_{L,z}}t_{2,2} t_{2,2}^{*}. \end{array} \end{aligned}$$

In the case of a junction along *x* axis, we can simply rewrite the scattering wave functions in different regions by interchanging $k_z$ and $k_x$ in the scattering wave functions given by Eqs. (8), (9) and (11), respectively. Moreover, the corresponding boundary conditions and definition of the transmission coefficients are obtained through Eqs. (12) and (13) by replacing $z\rightarrow x$ and $k_z\rightarrow k_x$, respectively. The solutions of *x* component of the wave vector are derived from the eigenvalue relation in WSM region given by Eq. (3) as follows,

14 $$\begin{aligned} k_{\pm ,x}=\sqrt{k_0^2-k_z^2-k_y^2-\frac{1}{2}\left( \frac{\gamma }{\mathcal {M}}\right) ^2 \pm \sqrt{\left( \frac{E-\mu _W}{\mathcal {M}}\right) ^2 -(k_0^2-k_y^2)\left( \frac{\gamma }{\mathcal {M}}\right) ^2+\frac{1}{4}\left( \frac{\gamma }{\mathcal {M}}\right) ^4}}. \end{aligned}$$

In the linear-response regime, the electrical and thermal currents passing through the junction are given respectively by^35^,

15 $$\begin{aligned} \begin{array}{c} I_{n}=\frac{e}{h}\mathop {\int }\limits _{0}^{\infty }dE T_n(E)[f_L(E)-f_R(E)], \\ {Q_{n}=\frac{1}{h}\mathop {\int }\limits _{0}^{\infty }dE (E-\mu ) T_n(E)[f_L(E)-f_R(E)],}\\ \\ T_n(E)=\mathop {\sum }\limits _{m=1}^{2}\mathop {\sum }\limits _{\textbf{k}_{\bot }}T_{m,n}(E,\textbf{k}_{\bot }), \end{array} \end{aligned}$$

where $T_n$, $I_{n}$ and $Q_{n}$ are total transmission probability, electrical and thermal currents for the electrons incident in the state $n=1,2$. In this equation $\textbf{k}_{\bot }$ denotes the transverse wave vector, $f_{L}(E)$ and $f_{R}(E)$ are Fermi distribution function of electrons in the left and right normal contacts, and $\mu$ is the chemical potential. In the continuum limit we can replace the summation over the transverse wave vector by an integration over it,

16 $$\begin{aligned} \mathop {\sum }\limits _{\textbf{k}_{\bot }}T_{m,n}(E,\textbf{k}_{\bot })= \frac{A}{(2\pi )^2}\left( \frac{E}{2\mathcal {M}}\right) \mathop {\int }\limits _{0}^{2\pi }d\varphi \mathop {\int }\limits _{0}^{\pi /2} T_{m,n}(E,\theta ,\varphi )\sin (2\theta )d\theta , \end{aligned}$$

where *A* denotes the cross section area of the junction, $\theta$ and $\varphi$ are the polar and azimuthal angles for the $\textbf{k}=(k_x,k_y,k_z)$.

Now, we consider that there is a voltage difference $\Delta V=(\mu _L-\mu _R)/e$, and temperature difference $\Delta \Theta =\Theta _L-\Theta _R$ between two normal contacts. For small values of $\Delta V$ and $\Delta \Theta$ we can apply a Teylor expansion for the distribution functions up to the first order of these quantities. As a result we find the electrical and thermal currents in terms of the linear electrical and thermoelectrical conductances $G_{n}$, $L_{n}$ and $K_{n}$ as follows,

17 $$\begin{aligned} \begin{array}{c} I_{n}=G_{n}\Delta V+L_{n}\Delta \Theta , \\ {Q_{n}=\Theta _0 L_{n}\Delta V+K_{n}\Delta \Theta ,}\\ \\ G_{n}=\frac{e^2}{h}\mathop {\int }\limits _{0}^{\infty }dE T_n(E)\left( -\frac{\partial f}{\partial E}\right) , \\ L_{n}=\frac{e}{h\Theta _0}\mathop {\int }\limits _{0}^{\infty }dE (E-\mu )T_n(E)\left( -\frac{\partial f}{\partial E}\right) , \\ {K_{n}=\frac{1}{h\Theta _0}\mathop {\int }\limits _{0}^{\infty }dE (E-\mu )^2T_n(E)\left( -\frac{\partial f}{\partial E}\right) ,} \end{array} \end{aligned}$$

where $\mu =(\mu _L+\mu _R)/2$ and $\Theta _0=(\Theta _L+\Theta _R)/2$ are common equilibrium chemical potential and temperature of the normal contacts. At low temperatures $G_{n}$, $L_{n}$ and $K_{n}$ reduce to the following equations using the Sommerfeld expansion^36^,

18 $$\begin{aligned} \begin{array}{c} G_{n}=\frac{e^2}{h}\mu \mathcal {T}_n(\mu ),\\ \\ L_{n}=\frac{e\pi ^2k_B^2\Theta _0}{3h}\left( \mathcal {T}_n(\mu )+\mu \frac{\partial \mathcal {T}_n(E)}{\partial E}\mid _{E=\mu }\right) ,\\ \\ {K_{n}=\frac{\pi ^2k_B^2\Theta _0}{3h}\mu \mathcal {T}_n(\mu ),} \end{array} \end{aligned}$$

where we have defined $\mathcal {T}_n(E)=T_n(E)/E$. Consequently, the total electrical and thermal currents are given by adding up the contribution of all accessible states for the incident electrons as follows,

19 $$\begin{aligned} \begin{array}{c} I=\mathop {\sum }\limits _{n=1}^{2} I_{n}=G\Delta V+L\Delta \Theta .\\ \\ {Q=\mathop {\sum }\limits _{n=1}^{2} Q_{n}=\Theta _0L\Delta V+K\Delta \Theta .} \end{array} \end{aligned}$$

The total electrical and thermoelectrical conductances are given by $G=\mathop {\sum }\nolimits _{n=1}^{2} G_{n}$, $L=\mathop {\sum }\nolimits _{n=1}^{2} L_{n}$ and $K=\mathop {\sum }\nolimits _{n=1}^{2} K_{n}$, respectively. Thermopower or Seebeck coefficient is defined as the voltage generated in the junction in response to a temperature difference under open circuite conditions, $S=(\Delta V/\Delta \Theta )_{I=0}$. From Eq. (19) we find that the Seebeck coefficient is simply given by,

20 $$\begin{aligned} S=-\frac{L}{G}=-\frac{L_{1}+L_{2}}{G_{1}+G_{2}}. \end{aligned}$$

Eventually, the electronic contribution to the thermal conductance is defined as the thermal current passing through the junction as a response to a temperature difference in the absence of the electrical current, $\kappa _{el}=(Q/\Delta \Theta )_{I=0}$.

21 $$\begin{aligned} \kappa _{el}=K-\Theta _0\frac{L^2}{G}=K-\Theta _0S^2G. \end{aligned}$$

The efficiency of a junction for presenting the thermoelectric effects are estimated by the thermoelectric figure of merit defined as follows,

22 $$\begin{aligned} ZT=\frac{\Theta _0GS^2}{\kappa _{T}}=\left( \frac{\kappa _{el}}{\kappa _{el}+ \kappa _{ph}}\right) \frac{\Theta _0L^2}{GK-\Theta _0L^2}, \end{aligned}$$

where $\kappa _{T}$ and $\kappa _{ph}$ are total and phononic contribution to the thermal conductance. In the following section, we calculate the electrical conductance *G*, thermoelectrical conductance *L*, Seebeck coefficient *S*, electronic contribution to the thermal conductance $\kappa _{el}$ and thermoelectric figure of merit *ZT* of the proposed junction in terms of its parameters. We only consider the electronic contribution to the thermal conductance. Since the phononic contribution to the thermal conductance is negligible at low temperatures $\kappa _{ph}\simeq 0$, it means that we investigate the low-temperature thermoelectric response of the proposed junction. Meanwhile, we have neglected the contribution of the Fermi arc surface states on the surface of WSM and only calculated the bulk states contribution in the thermoelectric coefficients. In fact, we can see that these states do not contribute at all to the thermoelectric properties of the junction along the *z* axis, and omitting their contribution and retaining the bulk states contribution for the junction along *x* axis is an excellent approximation for this junction (for details see the Supplementary Information).

## Results and discussions

In this section, we investigate the electronic and thermoelectric properties of N-WSM-N junction in terms of its parameters. We survey features of the junction along two perpendicular directions, one along the chiral axis (*z* axis) and the other perpendicular to the first one (*x* axis). Then, we compare the thermoelectric properties of the junction along these two perpendicular directions.

### N-WSM-N junction along the *z* axis

**Figure 2:**
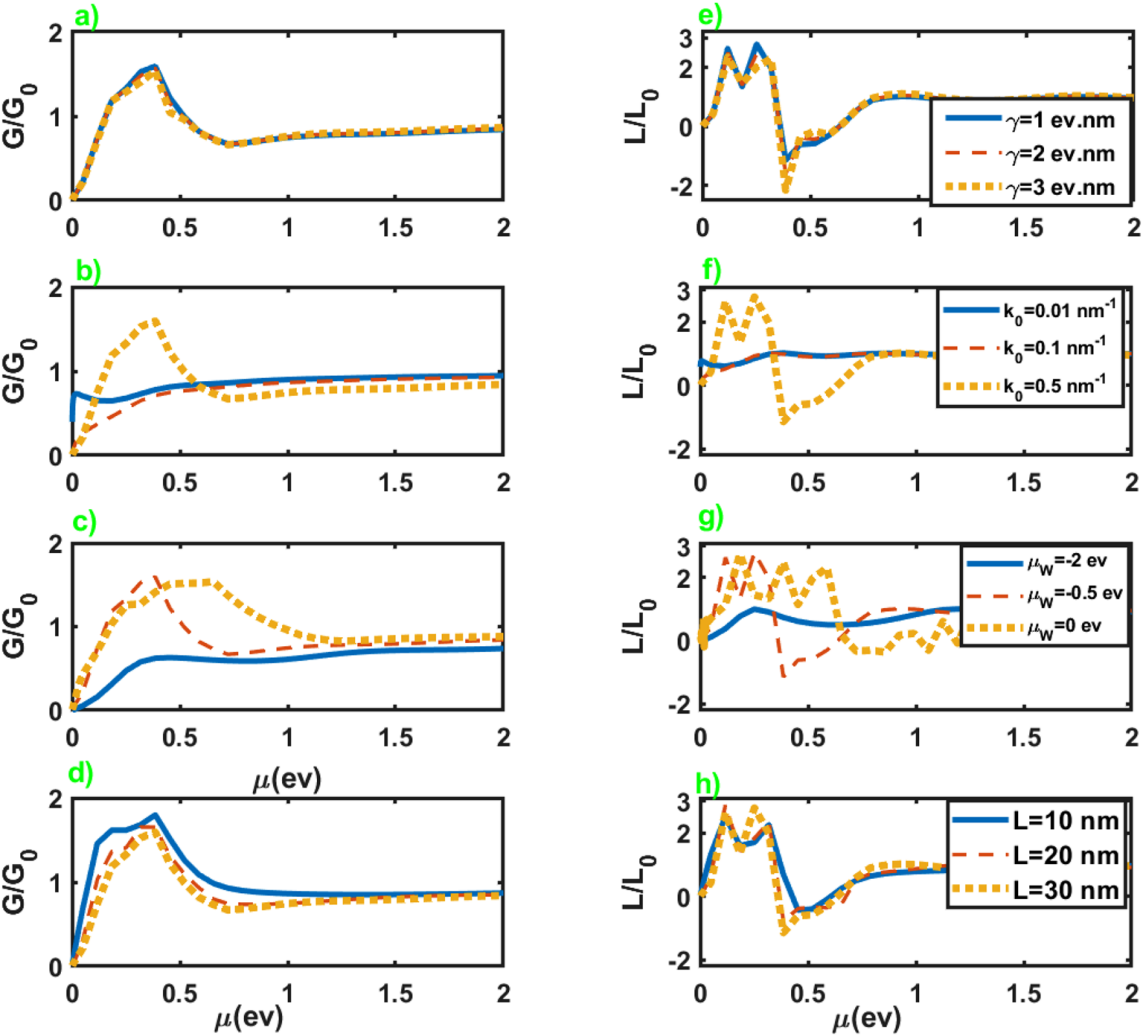
Normalized electrical conductance (left panel) and normalized thermoelectrical conductance (right panel) as a function of the chemical potential of the normal leads. The other parameters are $\mathcal {M}=5$ eV nm$^2$, $k_0=0.5$ nm$^{-1}$, $\mu _W=-0.5$ eV, $L=30$ nm for figures (**a**) and (**e**), $\mathcal {M}=5$ eV nm$^2$, $\gamma =1.0$ eV nm, $\mu _W=-0.5$ eV, $L=30$ nm for figures (**b**) and (**f**), $\mathcal {M}=5$ eV nm$^2$, $\gamma =1.0$ eV nm, $k_0=0.5$ nm$^{-1}$, $L=30$ nm for figures (**c**) and (**e**), $\mathcal {M}=5$ eV nm$^2$, $\gamma =1.0$ eV nm, $k_0=0.5$ nm$^{-1}$, $\mu _W=-0.5$ eV for figures (**d**) and (**h**).

First, we investigate the electrical and thermoelectric conductances of the junction along *z* axis. In Fig. 2, we have presented the normalized electrical conductance, $G/G_0$ with $G_0=(e^2/h)(\mu A/8\pi ^2\mathcal {M})$, and normalized thermoelectrical conductance, $L/L_0$ with $L_0=(e\pi ^2k_B^2\Theta _0/3h)(A/8\pi ^2\mathcal {M})$, in terms of the chemical potential of the normal leads for different values of the parameters of the junction. We can see that for high chemical potentials *G* and *L* show negligible dependence on the parameters of the junction, and considerable changes only happen at lower chemical potentials. As we can see, an increase in the length of the junction leads to a decrease in the electrical and thermoelectrical conductances. Nevertheless, increasing the chemical potential of WSM layer from negative values to zero leads to enhancement of them, in particular at lower chemical potentials. Moreover, an increase in the value of $k_0$ can substantially increase both conductances at lower chemical potentials, while they do not show considerable dependence on $\gamma$. It should be mentioned that the parameters $\gamma$ and $k_0$ are inherent characteristics of a WSM, and variation of these parameters generally means replacing the WSM sample with another one. Although, these parameters slightly change by imposing a strain on the WSM sample^37^. For more details about the electrical conductance see the supplementary information.

**Figure 3:**
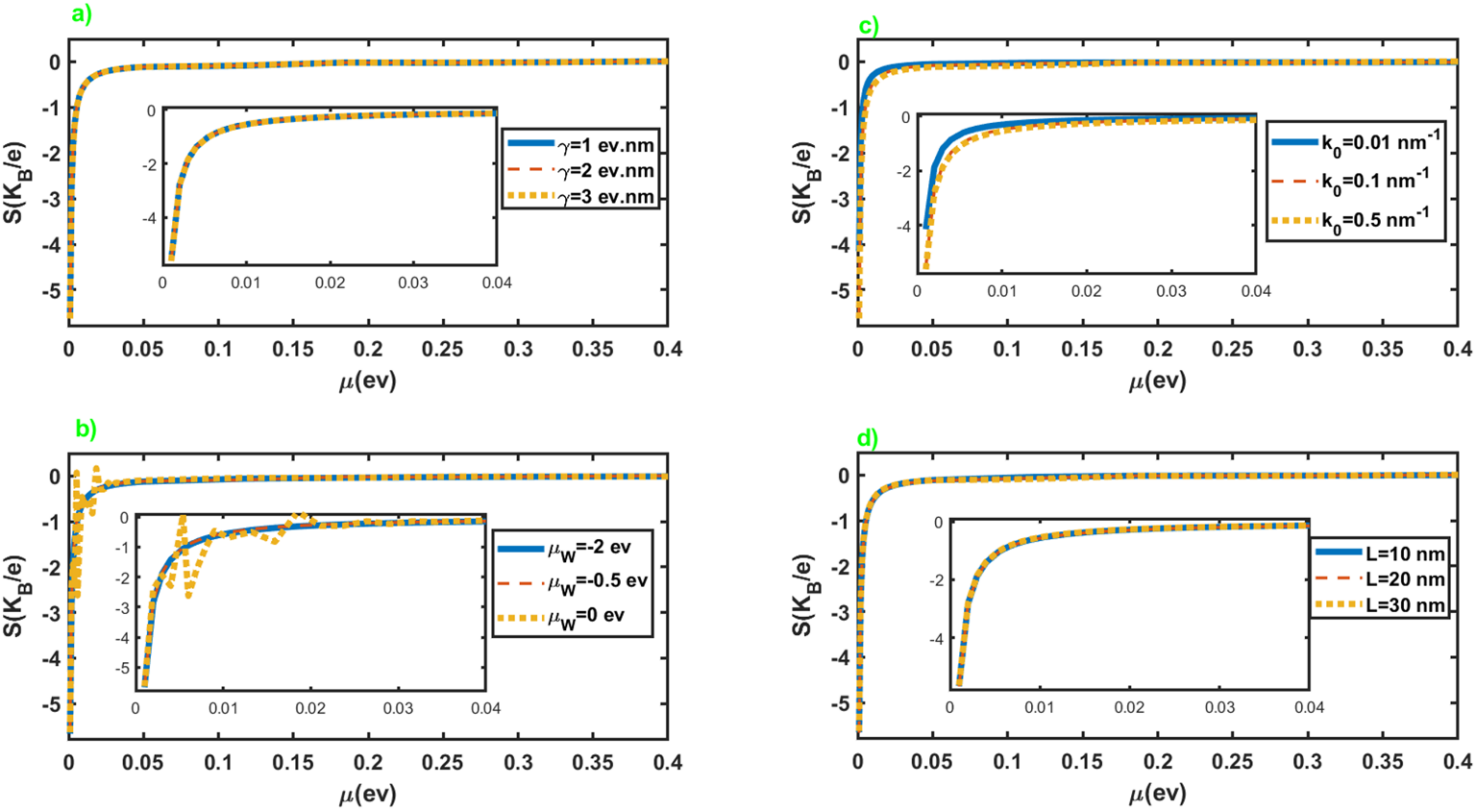
Seebeck coefficient as a function of the chemical potential of the normal leads for different values of the junction parameters. All of the other parameters are same as Fig. 2.

Figure 3 exhibits the Seebeck coefficient of the junction in terms of the chemical potential of the normal leads for different values of the parameters of the junction. As we can see from the figures, the Seebeck coefficient shows negligible dependence on the parameters of the junction. It can be attributed to the nearly similar effect of these parameters on the electrical and thermoelectric conductances, as is evident in Fig. 2. In addition, the application of the Sommerfeld approximation may remove small dependencies of the quantities on the parameters. Seebeck coefficient exhibits considerable values at very low chemical potentials where the conductance vanishes. Moreover, it diverges at vanishingly small chemical potentials, while it sharply approaches zero by increasing it. Furthermore, we do not observe a sign change in the seebeck coefficient by changing the chemical potential. It is reasonable for this junction since only electrons can involve in the thermoelectric effects.

**Figure 4:**
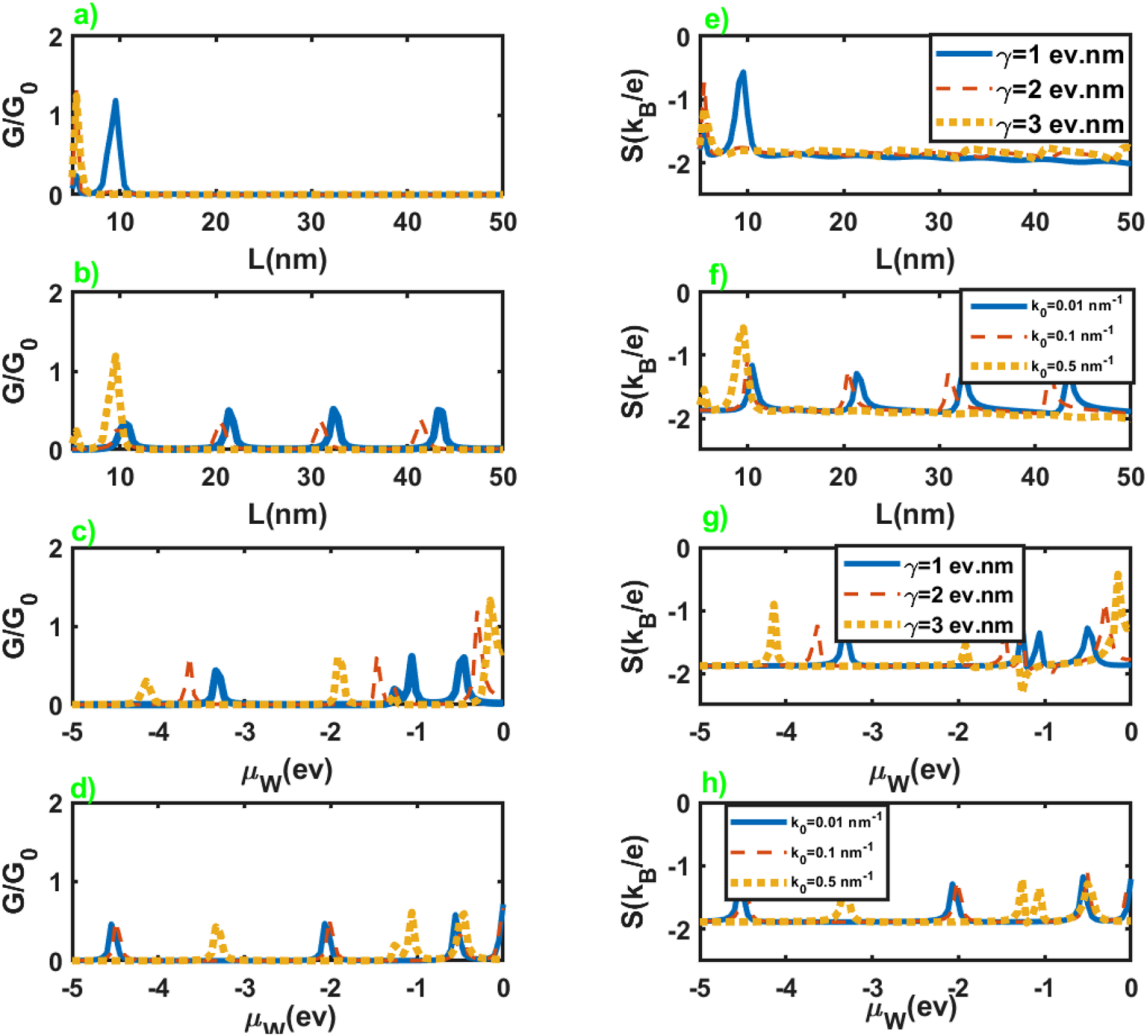
Electrical conductance (left panel) and Seebeck coefficient (right panel) as a function of the length (figures (**a**,**b**,**e**,**f**)), and chemical potential of the WSM layer (figures (**c**,**d**,**g**,**h**)) in terms of different values of $k_0$ and $\gamma$. Here $\mathcal {M}=5$ eV nm$^2$, $\mu =3.0$ meV and the values of the other parameters are considered as $k_0=0.5$ nm$^{-1}$, $\mu _W=-0.5$ eV for figures (**a**) and (**e**), $\gamma =1.0$ eV nm, $\mu _W=-0.5$ eV for figures (**b**) and (**f**), $k_0=0.5$ nm$^{-1}$, $L=30$ nm for figures (**c**) and (**g**), $\gamma =1.0$ eV nm, $L=30$ nm for figures (**d**) and (**h**).

We have presented dependence of the electrical conductance and Seebeck coefficient on the length and chemical potential of WSM layer in Fig. 4 in terms of the inherent properties of this layer, $\gamma$ and $k_0$, at very low chemical potential of the leads. As is clear from these figures, the conductance and Seebeck coefficient of the junction represent essential dependence on the length and chemical potential of WSM layer. On the other hand, we can infer that these parameters can serve as tuning parameters for electrical conductance and the Seebeck coefficient. Moreover, we see that conductance and Seebeck coefficient show nearly periodic peaks at the approximately common values of *L* and $\mu _W$.

**Figure 5:**
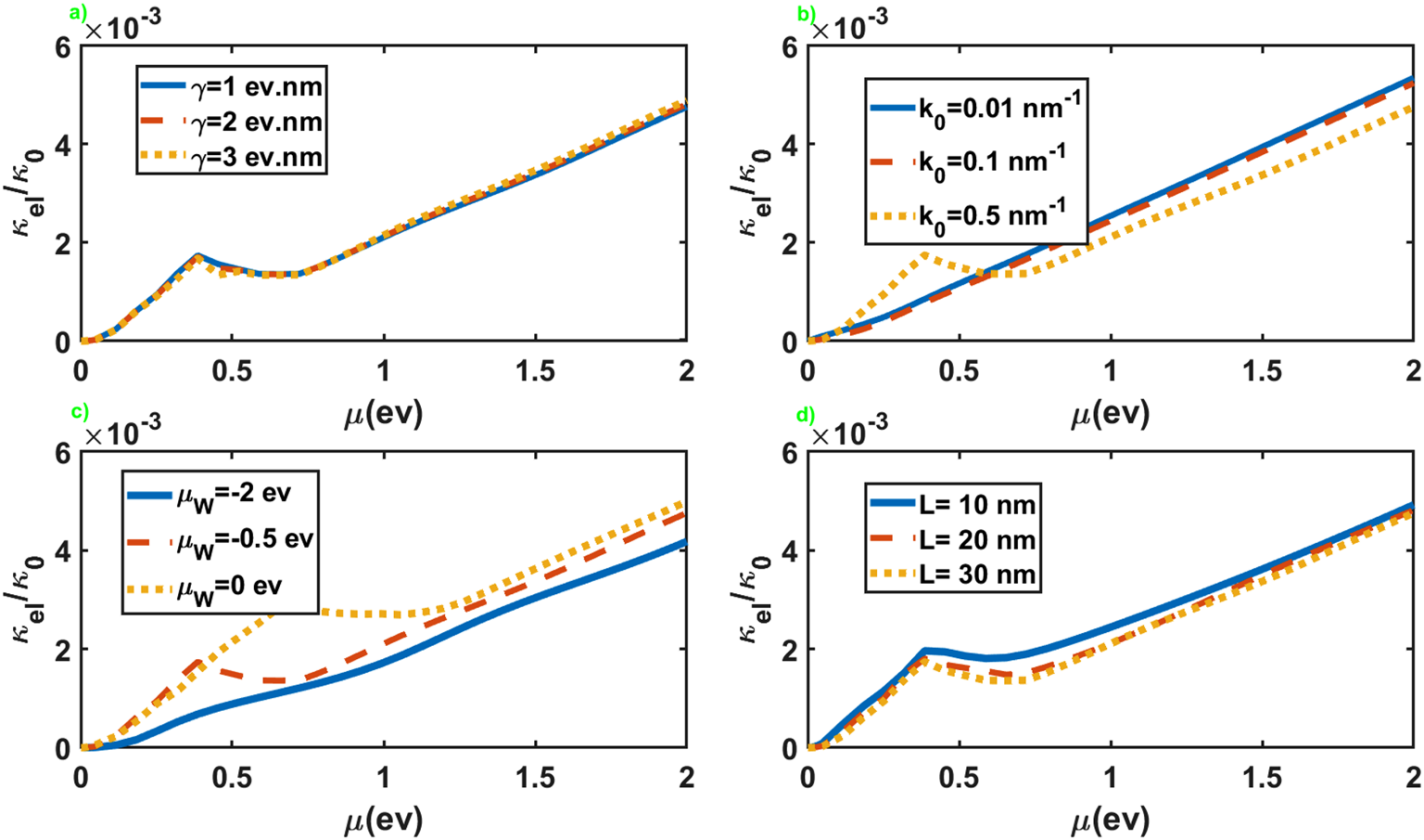
The electronic contribution to the thermal conductance as a function of the chemical potential of the normal leads for different values of the junction parameters. All of the other parameters are same as Fig. 2.

We have presented in Fig. 5 the normalized electronic contribution to the thermal conductance, $\kappa _{el}/\kappa _0$ with $\kappa _0=(\pi ^2k_B/3h)( A/8\pi ^2\mathcal {M})$, in terms of the chemical potential of the leads for different values of the other parameters. As we can see, $\kappa _{el}$ displays an increasing trend as a function of $\mu$, with a tiny slope at small chemical potentials and a nearly linear increase at large values of the chemical potential. For large values of $k_0$, it shows a peak and the chemical potential where this peak appears increases by increasing $\mu _W$. In addition, it presents little dependence on the values of $\gamma$, while it generally increases by increasing $\mu _W$ and decreases by increasing $k_0$ and *L*. As a result, we can adjust the electronic contribution to the thermal conductance by changing values of $\mu _W$ and *L* as the junction parameters.

**Figure 6:**
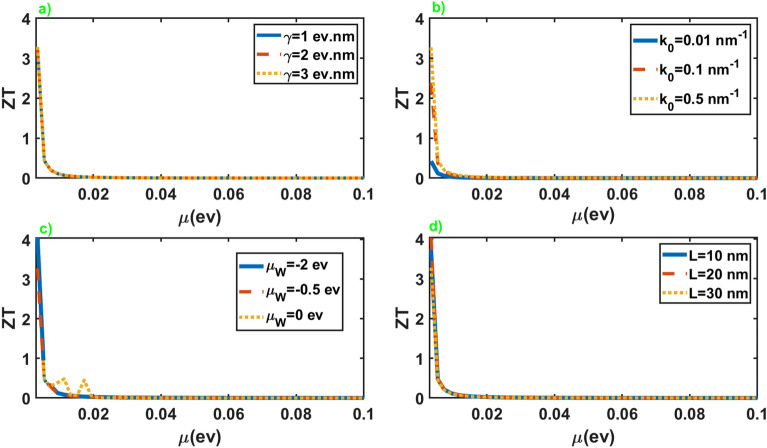
Thermoelectric figure of merit as a function of the chemical potential of the normal leads for different values of the junction parameters. All of the other parameters are same as Fig. 2.

Figure 6 exhibits the thermoelectric figure of merit of the junction in terms of the chemical potential of the normal leads. As is apparent, *ZT* represents extremely high values at small chemical potential of the normal leads and suppresses rapidly by increasing it. The appearance of high values for *ZT* originates essentially from the distinction in the electrical and thermal response of the junction at low chemical potentials of the leads, as can be seen in Figs. 2 and 5. In addition, *ZT* shows negligible dependence on the junction parameters at all chemical potentials except for small values. This extraordinarily high values of the thermoelectric figure of merit at low values of the leads chemical potential is vital for application in thermoelectric devices.

### N-WSM-N junction along the *x* axis

**Figure 7:**
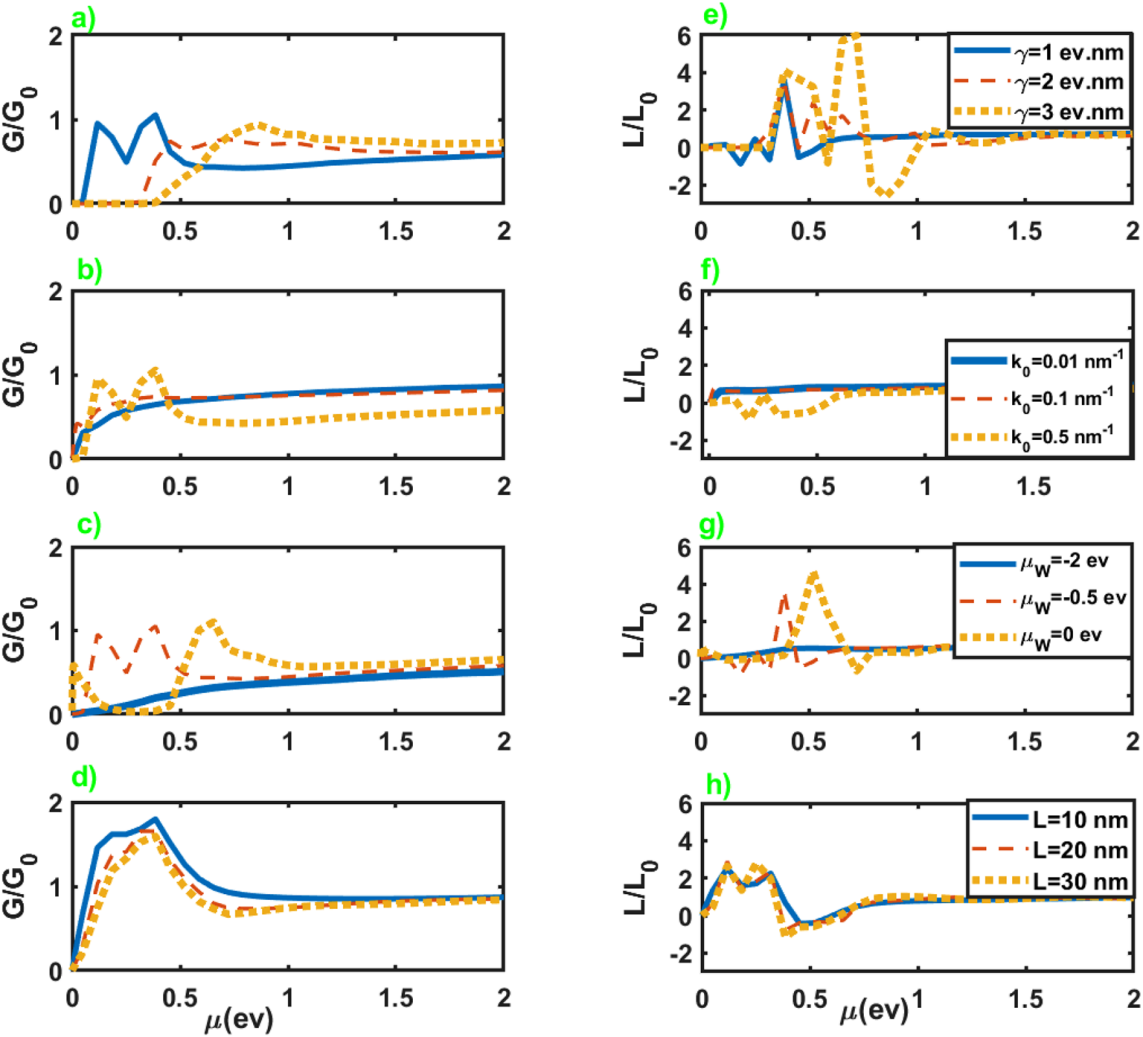
Normalized electrical conductance (left panel) and normalized thermoelectrical conductance (right panel) as a function of the chemical potential of the normal leads. All of the other parameters are same as Fig. 2.

Figure 7 represents normalized electrical and thermoelectrical conductances in terms of the chemical potential of the leads for different values of the junction parameters. An essential difference in the conductances of junctions along *z* and *x* axes is their considerable variation as a function of chemical potential at lower values in the last case in comparison to the former. Meanwhile, *L* represents higher variations by increasing $\gamma$ in the case of the junction along the *x* axis, while the variation of the other parameters approximately leads to the same values for *L* in both cases. For more details about the electrical conductance see the supplementary information.

**Figure 8:**
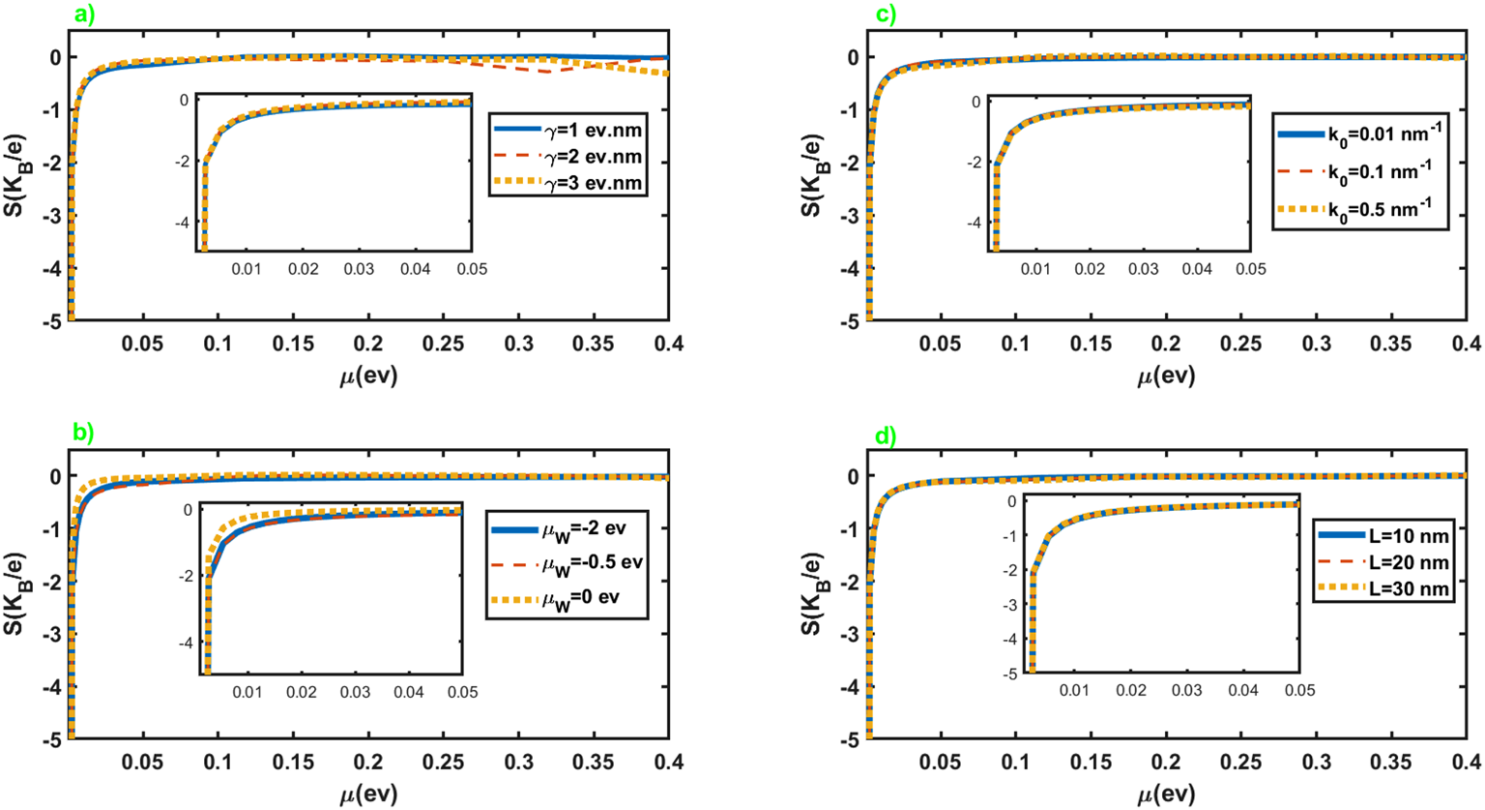
Seebeck coefficient as a function of the chemical potential of the normal leads for different values of the junction parameters. All of the other parameters are same as Fig. 2.

We have presented in Fig. 8 the Seebeck coefficient of the junction along *x* axis in terms of the chemical potential of the leads for different values of the junction parameters. The overall behavior is very similar to the case of the junction along the *z* axis. It diverges at vanishingly small chemical potentials, and by increasing the chemical potential, it suddenly drops to small values. Furthermore, it does not show considerable dependence on the junction parameters.

**Figure 9:**
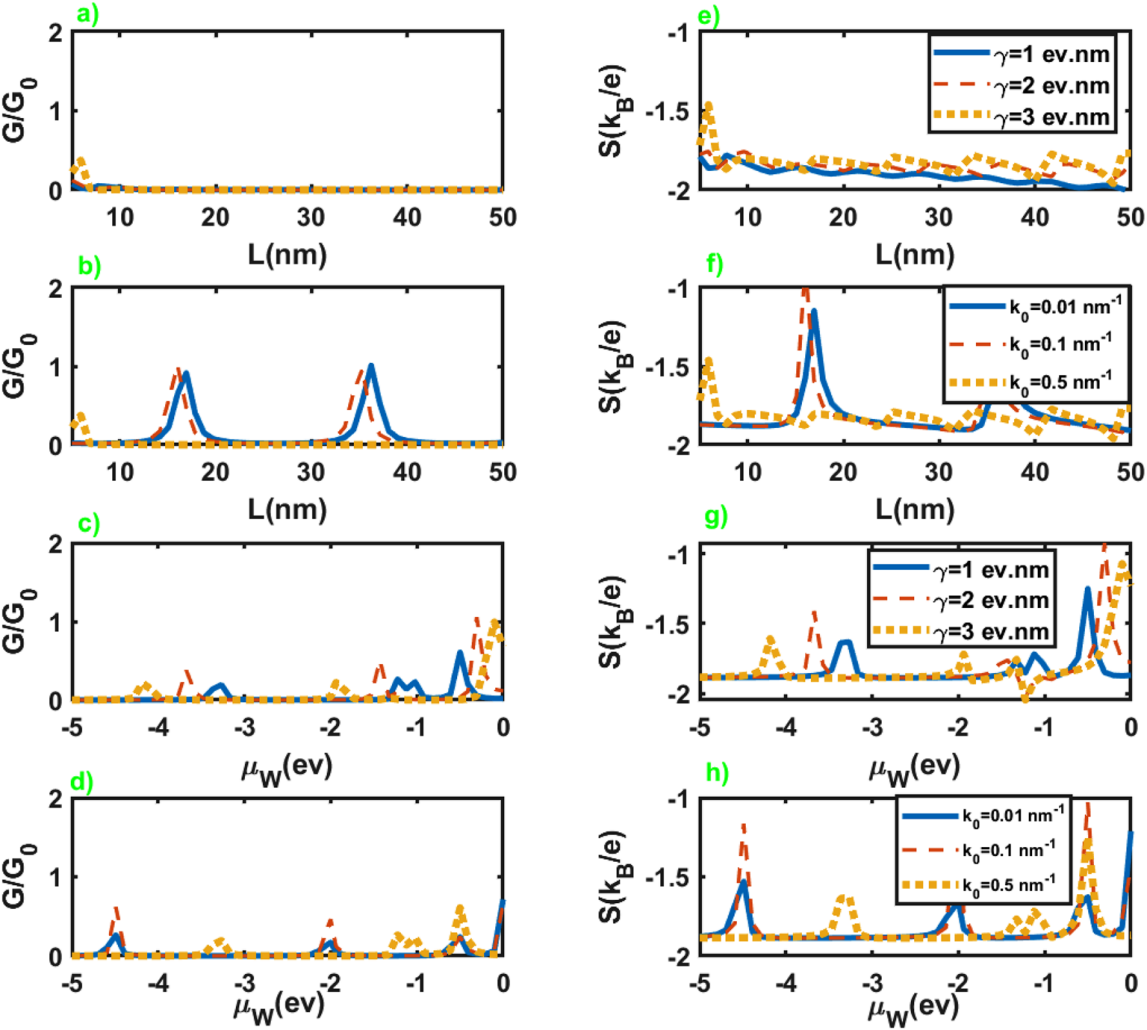
Electrical conductance (left panel) and Seebeck coefficient (right panel) as a function of the length (figures (**a**,**b**,**e**,**f**)), and chemical potential of the WSM layer (figures (**c**,**d**,**g**,**h**)) in terms of different values of $k_0$ and $\gamma$. All of the other parameters are same as Fig. 4.

The dependence of the electrical conductance and Seebeck coefficient on the length and chemical potential of WSM layer is exhibited in Fig. 9 for different values of $\gamma$ and $k_0$. As we can see in figures (a), (b), (e), and (f), conductance and Seebeck coefficient show a nearly oscillatory behavior in terms of the length of the junction. They show negligible dependence on the variation of $\gamma$, while change in the values of $k_0$ leads to the considerable variation in the conductance and Seebeck coefficient in terms of the length of the junction. Moreover, they show peaks in some values of $\mu _W$, and the hight of these peaks increases by increasing the value of $\gamma$ and $k_0$ as we can observe in figures (c), (d), (g) and (h).

**Figure 10:**
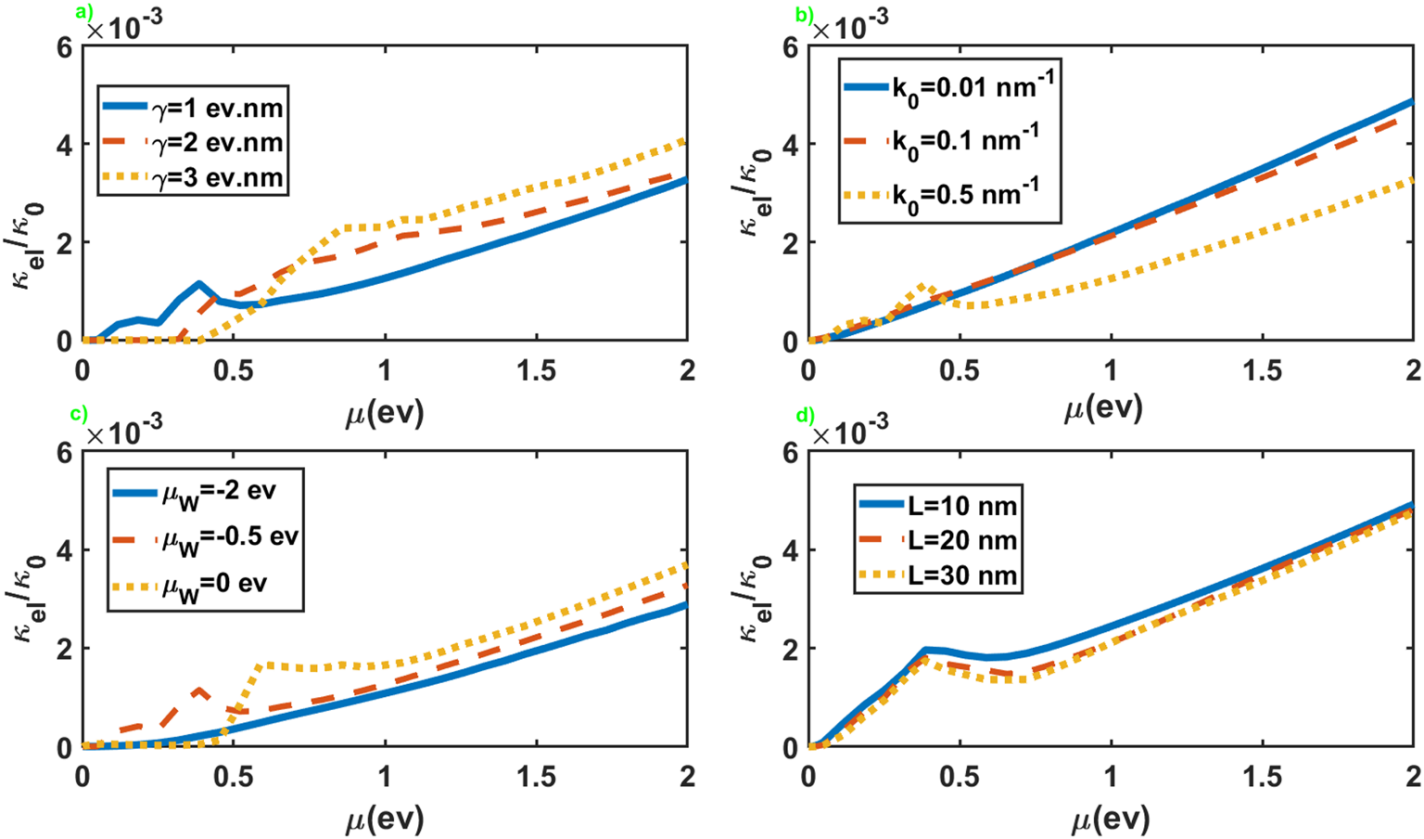
The electronic contribution to the thermal conductance as a function of the chemical potential of the normal leads for different values of the junction parameters. All of the other parameters are same as Fig. 2.

The normalized electronic contribution to the thermal conductance in terms of the chemical potential of the leads has been presented in Fig. 10 for the junction along the *x* axis. As we can see, the overall behavior of $\kappa _{el}$, in this case, is very similar to the junction along the *z* axis. The essential difference is the appearance of the threshold value for the chemical potential to maintain a nonzero thermal conductance in the junction along the *x* axis. This threshold chemical potential appears for large values of $\gamma$ and some values of $\mu _W$. Another significant difference is the substantial dependence of the thermal conductance on $\gamma$ in contrast to the former case.

**Figure 11:**
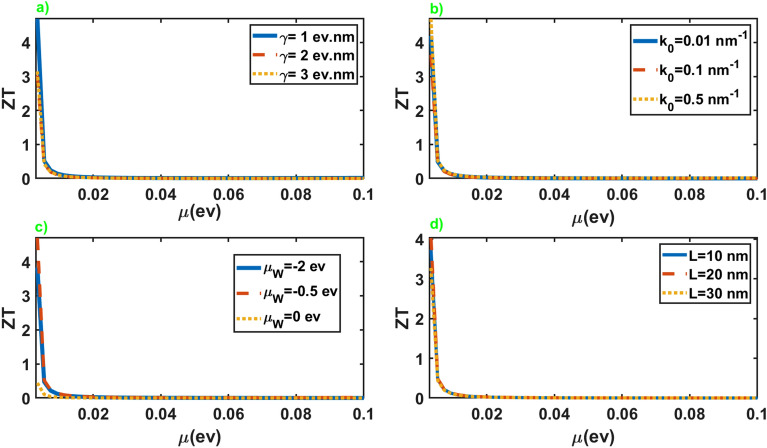
Thermoelectric figure of merit as a function of the chemical potential of the normal leads for different values of the junction parameters. All of the other parameters are same as Fig. 2.

Eventually, we put forward the results for the thermoelectric figure of merit of the junction along the *x* axis. Figure 11 illustrates the behavior of this quantity in terms of the chemical potential of the leads. As is apparent, the results are very similar to the case of the junction along the *z* axis. As before, the significant values of the figure of merit take place at the small chemical potentials of the leads. Nonetheless, this junction offers high figures of merit for both perpendicular directions. This intriguing results can be particularly noteworthy from a practical point of view.

## Conclusion

In summary, we have investigated the electronic and thermoelectric properties of a ballistic junction comprised of a WSM layer attached to two normal leads. We studied the properties of this junction in two different directions, one along the chiral axis of WSM and the other along the direction perpendicular to the first one. We found inherently direction-dependent electrical and thermal conductances for this junction originating from the anisotropic band structure of WSM. In the first case, electrical and thermal conductances show a broad peak in terms of the chemical potential of the leads, while in the second case, they represent a threshold for the chemical potential of the leads. In contrast to the conductances, the Seebeck coefficient and figure of merit exhibit approximately equivalent behavior in both directions. In particular, they reveal extremely high values at the small chemical potentials of the leads. According to these results, we can infer that this junction provides essentially direction-dependent and extremely high thermoelectric efficiency. These exciting properties demonstrate the high potential of this junction for application in thermoelectric devices.

We utilized a simplified version of the more accurate lattice Hamiltonian in our calculations, which describes all peculiarities of the inversion-symmetric WSMs very well. The reason for doing this is the complexities in dealing with the lattice Hamiltonian and the belief that it does not make a qualitative change in our results. Furthermore, this simplified Hamiltonian is exact at low energies, where the significant thermoelectric effects appear in the proposed junction. In addition, we have ignored the contribution of the Fermi arc surface states appearing on the surface of WSM in our investigation. However, we demonstrated in detail in the supplementary information that this contribution is negligible in contrast to the participation of the bulk states for the junction along *x* axis, and particularly, it becomes irrelevant for the *z* direction.

The experimental studies performed on the topological materials have revealed very high mobilities, even better than graphene, and long mean free paths of the order of $\sim 1\, \upmu m$ for this class of materials^38^. Hence, most of the current samples of WSMs can readily satisfy the ballistic conditions. Recently, a growing number of materials have been recognized as the magnetic or time-reversal breaking WSMs, such as $Co_2MnGa$^39^ and $Co_3Sn_2S_2$^40^, and so on. Since the obtained results for the Seebeck coefficient and thermoelectric figure of merit were not so sensitive to the inherent parameters of WSM, then they are valid for most of WSMs. Besides, we observe that the significant thermoelectric response of this junction takes place at small values of the chemical potential of the leads. To realize this condition experimentally, we need normal contacts possessing vanishingly small chemical potentials. Such feature can be satisfied by degenerate semiconductors with a relatively large gap, which allows for adjustment of the chemical potential via heavy doping^41^. Consequently, regarding the recent progresses in manufacturing multilayered structures composed of complex materials, the junction proposed in this article can be feasible in the experiment.

### Supplementary Information

Supplementary Information.

## Data Availability

All data generated or analysed during this study are included in this published article [and its supplementary information files].
