# Supplementary material for: Thermoelectric properties of ballistic Normal–Weyl semimetal-Normal junction
Source: Sci Rep. 2023 Aug 31;13:14263. doi: 10.1038/s41598-023-41355-3 (PMC10471620; doi:10.1038/s41598-023-41355-3)
Supplement: Supplementary file 1 — Supplementary Information. [file 41598_2023_41355_MOESM1_ESM.pdf]

Supplementary information for Thermoelectric properties of ballistic Normal-Weyl semimetal-Normal junction

Jafar Lotfi, Babak Abdollahipour‡

Faculty of physics, University of Tabriz, Tabriz 51666-16471, Iran

1. The electrical conductance

First, we investigate the electrical conductance of the junction along the chiral axis or z direction in terms of the different parameters characterizing it. Fig. S1 represents the normalized conductances, G_n/G_0 with $G_0 = (e^2/h)(\mu A/8\pi^2\mathcal{M})$, of the junction for electrons incident in two different states in terms of the chemical potential of the normal leads (μ) and distinct values of the other parameters of the junction. As we can see in these figures, the conductance of the first state G_1 shows an overall increase by increasing μ for all values of the other parameters of the junction, whereas the conductance of the second state G_2 vanishes at high chemical potentials after presenting a peak at small values. This behavior originates from the peculiarities of the junction of normal and WSM. Increasing the chemical potential of the normal leads cause the electronic dispersion of the first state in WSM to approach to the dispersion of the normal contacts, and it results in an increase in the conductance. While, for the second state difference in the energy dispersion in the normal contacts and WSM increases, which in turn leads to the suppression of the conductance.

Moreover, in Figs. S1 (d) and (h), we observe that the electrical conductance decreases for both states by increasing the length of the junction as we expect, while figures (c) and (g) show that increasing the chemical potential in WSM layer from negative values to zero leads to enhancement of conductance in both states and this effect is more considerable for the second state at low chemical potentials. In addition, we have analyzed the dependence of the conductance on the inherent parameters of WSM. We find that, by increasing value of γ, figures (a) and (e), the conductance of the first state decreases at high chemical potentials, while the conductance of the second state represents an increment. This feature arises since parameter γ indicates the strength of mixing between two states inside the WSM segment. Therefore, by increasing the value of γ, two states mix increasingly and this leads to decreasing the conductance of the first state and an increment of the conductance of the second state. Meanwhile, by increasing the value of k_0 which denotes the separation of the Weyl nodes

‡ Corresponding author: b-abdollahi@tabrizu.ac.ir

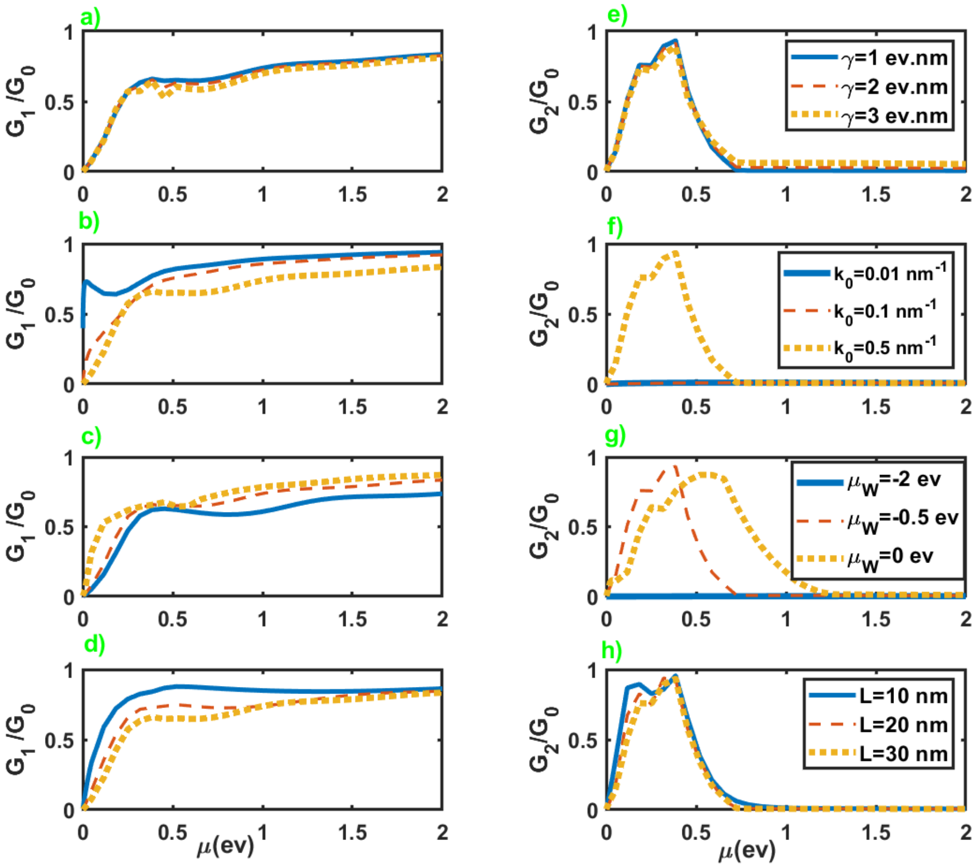

Figure S1. Normalized conductances of first and second states for the junction along z axis as a function of the chemical potential of normal leads for different values of γ depicted in a) and e), k_0 in b) and f), μ_W in c) and g), L in d) and h). The other parameters are $\mathcal{M} = 5eV.nm^2$, $k_0 = 0.5nm^{-}1$, $\mu_W = -0.5eV$, $L = 30nm$ for figures a) and e), $\mathcal{M} = 5eV.nm^2$, $\gamma = 1.0eV.nm$, $\mu_W = -0.5eV$, $L = 30nm$ for figures b) and f), $\mathcal{M} = 5eV.nm^2$, $\gamma = 1.0eV.nm$, $k_0 = 0.5nm^{-}1$, $L = 30nm$ for figures c) and e), $\mathcal{M} = 5eV.nm^2$, $\gamma = 1.0eV.nm$, $k_0 = 0.5nm^{-}1$, $\mu_W = -0.5eV$ for figures d) and h).

we observe a suppression in conductance of the first state at all chemical potentials, figure (b), whereas conductance of the second state exhibits a considerable enhancement by increasing k_0 as it is clear from figure (f).

Now we analyze the behavior of the electrical conductances of N-WSM-N junction for the first and second states when the junction direction is perpendicular to the chiral axis or along the x axis. As we can see in Fig. S2, for the higher chemical potentials of the leads, we observe a behavior similar to the junction along the z axis when γ, k_0, the chemical potential and length of the WSM layer vary. However, in this case, conductance shows a more profound dependence on the junction parameters in comparison to the junction along z axis. In particular, the dependence of the conductance in both states on γ has been considerably enhanced in the case of the junction along the x axis. We find that there is a chemical potential threshold for the conductance of both states, which increases by increasing the value of γ. Moreover, by increasing γ conductance of both

states decrease (increase) at lower (higher) chemical potentials. While we observe an increase (decrease) in the conductance of both states by increasing k_0 at lower (higher) chemical potentials. By increasing values of μ_W, a peak is generated in the conductance of both states at very low chemical potentials. Finally, we find that the conductance of both states has identical behavior in both junctions.

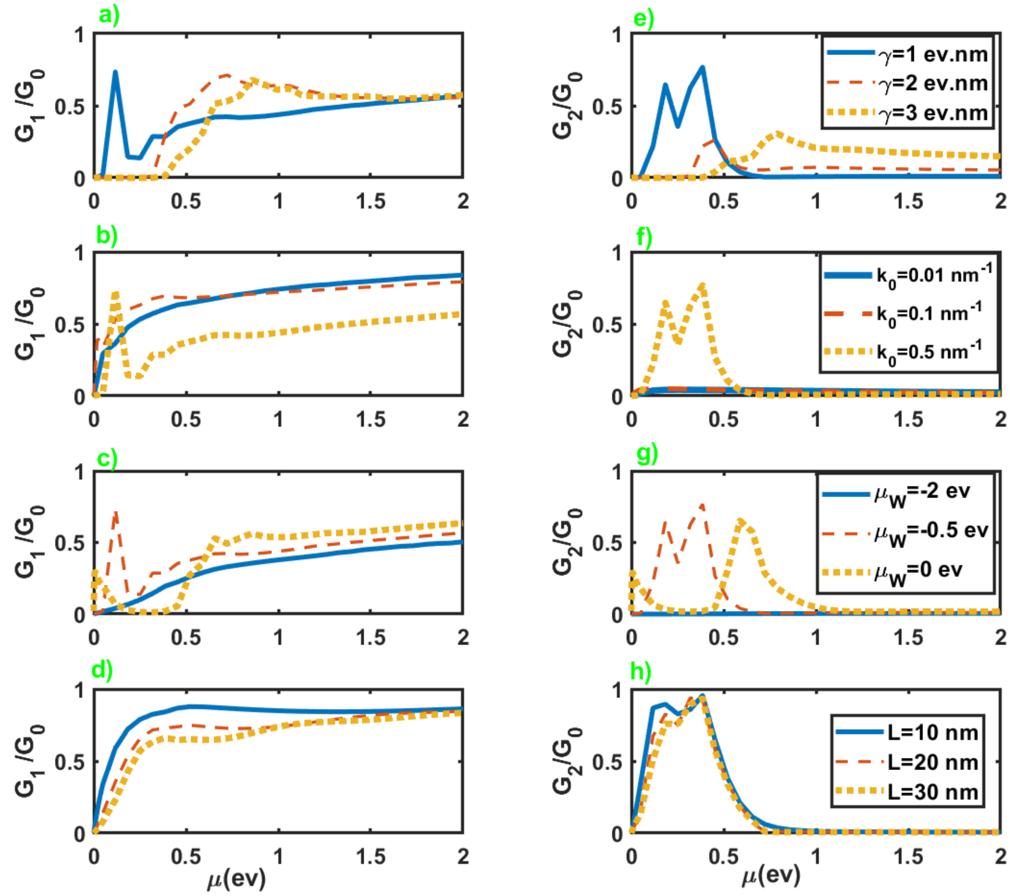

Figure S2. Normalized electrical conductances of the first and second states for the junction along x axis as a function of the chemical potential of the normal leads. The values of other parameters are identical with Fig. S1.

2. Contribution of the Fermi arc surface states

In a finite WSM, edge states manifest themselves as the Fermi arc states on the surface of the relevant Brillouin zone, which connect the projections of the Weyl-fermion Fermi surfaces in momentum space [1]. According to the minimal model of WSM, the surface states appear on the terminated faces of the WSM layer along the x and y axes [2]. The corresponding Fermi arcs emerge on the relevant surfaces of the Brillouin zone and parallel to the k_z direction in the momentum space. It has been shown that these states are described by the Hamiltonians $\mathcal{H}_{s,x} = \gamma k_y$ and $\mathcal{H}_{s,y} = \gamma k_x$ [2], respectively. Therefore, the Fermi velocities of these surface states are along the x and y directions,

perpendicular to the z axis. As a result, we conclude that these surface states can not contribute to the electrical and thermal currents of the junction along the z axis. In the following, we concentrate on the contribution of the Fermi arc surface states in the thermoelectric coefficients of the junction along the x axis.

We consider the Fermi arc surface states on the surfaces of WSM denoted by $y = 0$ and $y = W$, where W denotes the thickness of the layer along the y axis. Electrons in the surface states appearing on two opposite faces propagate in opposite directions without backscattering. For an almost thick layer of WSM, $100nm < W < 1\mu m$, we can suppose the following form for the wave function of the surface states on the face $y = 0$,

$$\psi_{s,\lambda} = \begin{pmatrix} s_1 \\ s_2 \end{pmatrix} e^{ik_x x + ik_z z} e^{-\lambda y}. \tag{1}$$

Applaying this wave function to the bulk Hamiltonian given by Eq. 1 in the main text and imposing the relevant boundary conditions to have zero wave function at this surface, the corresponding values of the decay constant are obtained λ as follows [2],

$$\lambda^2 = -\kappa^2 + \frac{\gamma^2 \pm \sqrt{4\mathcal{M}^2 E^2 - 4\mathcal{M}^2\gamma^2(\kappa^2 + k_x^2) + \gamma^4}}{2\mathcal{M}^2}, \tag{2}$$

where $\kappa^2 = k_0^2 - k_x^2 - k_z^2$, $k_x^2 + k_z^2 < k_0^2$ and $E = \gamma k_x$. Due to the gapless band structure of the bulk states in WSM, the bulk and surface states are completely coupled in the ballistic regime, and we can not express the contribution of these states in the electrical or thermal currents by separate expressions. Nevertheless, in the following, we will argue that in the proposed junction and for a relatively thick WSM sample, $W >> \lambda$, the contribution of the surface states in the electrical and thermal conductances is negligible relative to the contribution of the bulk states. To show it, we remember that normal contacts do not support surface states, and only bulk states can propagate in the normal contacts. Thus, the wave function for an incident electron in the normal side along the x axis can be written as,

$$\psi_N = \begin{pmatrix} c_1 \\ c_2 \end{pmatrix} e^{ik_x x + ik_z z} \sin(k_y y), \tag{3}$$

where $k_y, k_z < \sqrt{E/M}$, and wave vector along the y axis takes discreet values equal to $k_y = n\pi/W$. The wave function for the surface state on the $y = 0$ surface can be written as [2],

$$\psi_{s,\lambda} = \begin{pmatrix} s_1 \\ s_2 \end{pmatrix} e^{ik_x x + ik_z z} \left(e^{-\lambda_1 y} - e^{-\lambda_2 y}\right), \tag{4}$$

where $\lambda_{1,2}$ are solutions for the decay constant given by Eq. 2. The amplitude of transition from a bulk state in the normal side to a bulk state in WSM can be written as,

$$A_{b\to b} = \int_0^W dy \sin(k_y y)\sin(k_y y) = \frac{W}{2} - \frac{\sin(2k_y W)}{4k_y}, \tag{5}$$

by setting $k_y = n\pi/W$ the result reduces to $W/2$. However, the amplitude of transition from an incident bulk state in the normal side to a surface state in WSM is given by,

$$A_{b\to s} = \int_0^W dy \sin(k_y y)\left(e^{-\lambda_1 y} - e^{-\lambda_2 y}\right) = \frac{e^{-W\lambda_1}\left(e^{W\lambda_1}k_y - k_y\cos(k_y W) - \lambda_1 \sin(k_y W)\right)}{k_y^2+\lambda_1^2} - \frac{e^{-W\lambda_2}\left(e^{W\lambda_2}k_y - k_y\cos(k_y W) - \lambda_2 \sin(k_y W)\right)}{k_y^2+\lambda_2^2}. \quad (6)$$

Now if we impose the condition of thick sample $W >> \lambda_1, \lambda_2$ the result reduces to,

$$A_{b\to s} = \frac{k_y(\lambda_2^2-\lambda_1^2)}{(k_y^2+\lambda_1^2)(k_y^2+\lambda_2^2)}, \quad (7)$$

$$\lambda_2^2-\lambda_1^2 = 2\sqrt{\left(\frac{E}{\mathcal{M}}\right)^2 - \left(\frac{\gamma}{\mathcal{M}}\right)^2(k_0^2+k_z^2) + \frac{1}{4}\left(\frac{\gamma}{\mathcal{M}}\right)^4}. \quad (8)$$

For small values of $E << \mathcal{M}$, which we are interested, we have,

$$\lambda_2^2-\lambda_1^2 \simeq \frac{\gamma}{\mathcal{M}}\sqrt{\left(\frac{\gamma}{\mathcal{M}}\right)^2 - 4(k_0^2+k_z^2)}. \quad (9)$$

We can see that for the typical values of the parameters considered in our calculation, $\mathcal{M} = 5eV.nm^2$, $\gamma = 1.0eV.nm$, $k_0 = 0.5nm^-1$, and for a small value of the chemical potential $E \simeq \mu \simeq 0.05eV$, we find that $\lambda_2^2 - \lambda_1^2 \simeq 0.2i$. Thus, we expect that the probability of transition between the bulk states in normal and the surface states in the WSM be of the order of $|A_{b\to s}|^2 \simeq 0.001$. The ratio of the probability of transition from bulk to surface state to the probability of transition from bulk to bulk state is given approximately by $|A_{b\to s}|^2/|A_{b\to b}|^2 \simeq 0.004/W^2$, which is negligible for a reasonably thick layer of WSM, $W \sim 100-1000nm$. To illustrate this argument, we have plotted in Fig. S3 the probability of transition from bulk to surface state as a function of the chemical potential of the leads, for the values of the parameters stated above and $k_y = 0.1\sqrt{\mu/\mathcal{M}}$. This plot shows small values for the transition probability in the interested range of the chemical potential. Moreover, Fig. S4 represents this transition probability in terms of the k_y in the full range for $\mu = 0.05eV$. Again, we find small values for this ratio for all possible values of k_y.

Consequently, we observe that in the proposed junction the contribution of the surface states in the electrical and thermal currents is insignificant relative to the bulk states. On the other hand, depending on the junction direction, some surface states may lie at the interface of the normal and WSM. Once more, it has been shown that due to the localization of these states on the interface and possessing Fermi velocities parallel to the interface, they can not contribute to the scattering processes and, hence, in the longitudinal current (current normal to the interface) [3]. Therefore, according to the above argument, we infer that by a good approximation, we can disregard the contribution of the surface states in the thermoelectric properties of the proposed

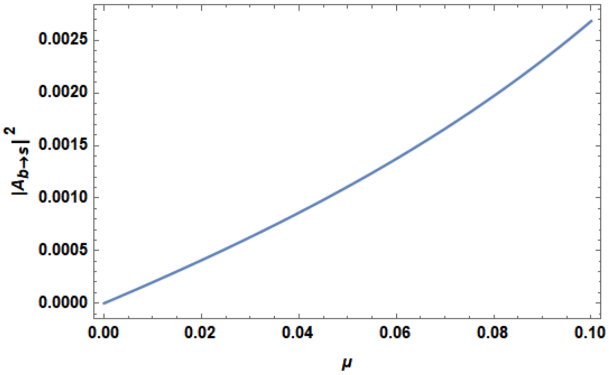

Figure S3. Probability of transition form a bulk state in the normal contact to a surface state in the WSM layer as a function of the chemical potential of the normal leads for $k_y = 0.1\sqrt{\mu/\mathcal{M}}$.

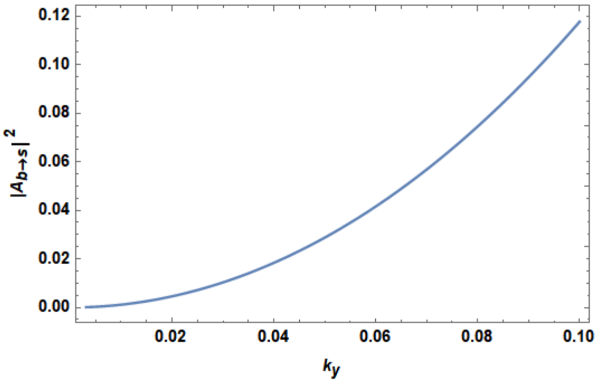

Figure S4. Probability of transition form a bulk state in the normal contact to a surface state in the WSM layer as a function of k_y for $\mu = 0.05$.

junction. Besides, due to the gapless spectrum of the bulk and Fermi arc surface states in WSM, and termination of the lower limit of the energy of the incident electrons by the normal contacts, the contribution of the Fermi arc states in the thermoelectric coefficients of the proposed junction will be additive with the contribution of the bulk states. As a result, our main results will not change qualitatively by including the effect of the Fermi arc states, and we expect their contribution cause enhancement of the observed high values of the Seebeck coefficient and thermoelectric figure of merit.

3. References

[1] N. P. Armitage, E. J. Mele and A. Vishwanath, Weyl and Dirac semimetals in three-dimensional solids, Rev. Mod. Phys. **90**, 015001 (2018), DOI: https://doi.org/10.1103/RevModPhys.90.015001

[2] G. Chen, O. Zilberberg, and W. Chen, Detection of Fermi arcs in Weyl semimetals through surface negative refraction, Phys. Rev. B **101**, 125407 (2020). DOI: https://doi.org/10.1103/PhysRevB.101.125407

[3] S. Uchida, T. Habe, and Y. Asano, Andreev Reflection in Weyl Semimetals, J. Phys. Soc. Jpn. **83**, 064711 (2014). DOI: https://doi.org/10.7566/JPSJ.83.064711
